# Manipulation of genes could inhibit SARS-CoV-2 infection that causes COVID-19 pandemics

**DOI:** 10.1177/15353702211008106

**Published:** 2021-04-25

**Authors:** Arnab Banerjee, Sandip Mukherjee, Bithin K Maji

**Affiliations:** Department of Physiology (UG & PG), Serampore College, Serampore, Hooghly 712201, India

**Keywords:** COVID-19, SARS-CoV-2, viral, CRISPR/Cas system, PAC-MAN, antiviral strategy

## Abstract

The year 2020 witnessed an unpredictable pandemic situation due to novel coronavirus (COVID-19) outbreaks. This condition can be more severe if the patient has comorbidities. Failure of viable treatment for such viral infection caused by severe acute respiratory syndrome coronavirus 2 (SARS-CoV-2) is due to lack of identification. Thus, modern and productive biotechnology-based tools are being used to manipulate target genes by introducing the clustered regularly interspaced short palindromic repeats (CRISPR)/Cas (CRISPR-associated) system. Moreover, it has now been used as a tool to inhibit viral replication. Hence, it can be hypothesized that the CRISPR/Cas system can be a viable tool to target both the SARS-CoV-2 genome with specific target RNA sequence and host factors to destroy the SARS-CoV-2 community via inhibition of viral replication and infection. Moreover, comorbidities and COVID-19 escalate the rate of mortality globally, and as a result, we have faced this pandemic. CRISPR/Cas-mediated genetic manipulation to knockdown viral sequences may be a preventive strategy against such pandemic caused by SARS-CoV-2. Furthermore, prophylactic antiviral CRISPR in human cells (PAC-MAN) along with CRISPR/Cas13d efficiently degrades the specific RNA sequence to inhibit viral replication. Therefore, we suggest that CRISPR/Cas system with PAC-MAN could be a useful tool to fight against such a global pandemic caused by SARS-CoV-2. This is an alternative preventive approach of management against the pandemic to destroy the target sequence of RNA in SARS-CoV-2 by viral inhibition.

## Impact statement

Nowadays, manipulation of genes is the most potent weapon against some deadly viral-mediated infections. CRISPR/Cas system is one of them which act as a molecular scissor to impede viral replication caused by SARS-CoV-2. This tool directly targets the specific RNA sequence and thereby knocks down specific lines to inhibit replication and viral infection.

## Introduction

SARS-CoV-2 causes the COVID-19 pandemics; in China, people could have been infected due to the use of seafood from China’s local market and also due to the exposure to infected animals. On the contrary, in some cases, investigations showed that people with no previous record of visiting the seafood market or exposed to infected animals in the market were also infected. Further, the virus spread over the population via sneezing, coughing, and aerosols from one person to another, leading to a severe infection of the lungs through the nasal cavity or mouth; until now, seven types of coronaviruses caused infection in humans.^1^ The severe acute respiratory syndrome coronavirus and Middle East respiratory syndrome coronavirus were originated from a zoonotic population in humans in 2003 and 2012, respectively.^2^ Among other coronavirus strains, the seventh one is severely contagious SARS-CoV-2 which causes severe respiratory disease and the current pandemic situation worldwide. The positive-sense RNA virus SARS-CoV-2 mainly infects the upper and lower part of the tracks of the respiratory system of humans.^3^ However, coronavirus infection alters the redox-status by generating reactive oxygen species (ROS) followed by oxidative stress (OS) by manipulating host cell machinery, which causes different abnormal situations in the human body.^4,5^ Moreover, regular consumption of a high-lipid diet alone or monosodium glutamate also disturbs the redox-equilibrium;^6,7^ so it can hypothesize that such types of diets cause OS, which may indirectly stimulate the viral infection. Therefore, it can predict that OS plays a pivotal role in SARS-CoV-2 disorders that alter redox signaling to increase the severe acute respiratory syndrome risk. As there is no such effective treatment for the condition of contagious SARS-CoV-2, the best way to stop the pandemic is to find a possible effective antiviral strategy of management against COVID-19. The present review offers the development of the antiviral prophylactic strategy against SARS-CoV-2 via manipulation of genes by the CRISPR/Cas system.

## CRISPR/Cas system

Nowadays, genetic engineering-based work makes it researchers and scientists easy to trace the master regulator of any disease and treat them accordingly. The CRISPR/Cas system has been found in most prokaryotes such as bacteria and archaea, conferring adaptive immunity against invading nucleic acids. CRISPR/Cas is a molecular tool in genetic engineering and helps to identify the target genes in various diseases. Besides editing deoxyribonucleic acid (DNA), they have also played a crucial role in editing and target specific ribonucleic acid (RNA) sequences. It also acts as a DNA/RNA-guided/targeting tool in prokaryotic organisms with inherited adaptive immunity to combat against xenobiotics.^8^ The systems have two classes; the class 1 system includes type I, III, and IV CRISPR/Cas system, which uses crRNA with multi-effector complex to find out the target sequence and then cleave them. On the other hand, class 2 includes type II/Cas9, type V/Cas12, and type VI/Cas13, which uses a single multi-domain Cas protein with CRISPR RNA (crRNA) for involvement.^8^ The second class of CRISPR/Cas system has been vital for genomic editing and diagnostic markers of different infections.^9–12^ On the contrary, recently, CRISPR/Cas12a, CRISPR/Cas13a, and CRISPR/Cas13b have been utilized to establish an accurate diagnostic tool to detect bacteria and virus-mediated infections in humans.^12–14^ Recently, molecular tool-based technology is being used to fight against various bacteria or virus-mediated infections by modifying gene sequences. Therefore, we hypothesize that it may be a viable tool to combat against SARS-CoV-2-mediated infection in the body.^15^

## CRISPR/Cas system versus human pathogenic viruses

Apart from targeting human genomic DNA, the “molecular scissors” (i.e. CRISPR/Cas system) can also target any double-stranded viral invaders in humans’ case. There is a plethora of evidence where the CRISPR/Cas technology inhibits the number of viral infections in human *in vitro* and *in vivo* models.^16^ CRISPR/Cas technology has a potential antiviral strategy against human immunodeficiency virus (HIV), Epstein-Barr virus (EBV), Herpes simplex virus (HPV), hepatitis B virus (HBV), hepatitis C virus (HCV), human papillomavirus (HPV), human polyomaviruses or John Cunningham virus (JCV), human cytomegalovirus (HCMV), African swine fever virus (ASFV), and Pseudorabies virus (PV) most likely mediated via inhibition of the translation, replication of the viruses, or direct disruption of the viral genome.^17^ Moreover, in the case of HCV, this single-stranded RNA virus causes inflammation of liver and hepatocytes infection worldwide. CRISPR/Cas plays an anti-viral tool to disrupt the genome of the HCV to combat against the HCV-mediated infections. In this case, the earlier study suggested that the potential power of the Cas9 from *Francisella novicida* (FnCas9) targets mRNA of bacteria and induced repression of viral genes. Therefore, the CRISPR/FnCas9 system’s antiviral strategy via targeting the RNA to fight against HCV infection in eukaryotic organisms was unique. Previously, researchers also pointed out the HCV translation inhibition by inhibition of D11A/H969A (the catalytically inactive version of FnCas9)^17^. Therefore, there is no need for direct degradation of RNA in the virus for the stoppage of translation of viral protein by the CRISPR/FnCas9 system. Alternatively, the connection of FnCas9 with the RNA of HCV genome appeared to be sufficient for inhibition of the translation and replication of the virus. Hence, it can infer that the specification of applying CRISPR/Cas in case of RNA viruses seems to be little bit different from that of DNA viruses.

## SARS-CoV-2 and pathogenesis

The coronavirus consists of various proteins such as spike protein, membrane, envelope, and nucleocapsid. The heavily glycosylated spike protein (type I) present on the external surface is attached with the host angiotensin-converting enzyme two receptors (ACE2R).^18^ This glycosylated type I protein is a valuable determinative of the SARS-CoV-2 insertion into the host cells as the receptor-binding domain for the ACE2R, and direct membrane fusion plasma membrane with the SARS-CoV-2 that is present on the head of the virus.^19,20^ Besides that, SARS-CoV-2 also gets entry to the host cells via clathrin-dependent and clathrin-independent endocytosis.^21,22^ Subsequently, the RNA genome of SARS-CoV-2 released into the cytoplasm, translated into two polyproteins and structural proteins to begin the replication of the viral genome.^23^ Primarily, polyprotein 1a/1ab is translated from the genomic RNA, which ultimately constructs the replication-transcription complex in a double-membrane vesicle after the infection of SARS-CoV-2. Eventually, in a discontinuous manner, the copy numbers of RNAs are increased by the replication-transcription complex. Additionally, the viruses containing the vesicles are fused with the plasma membrane to liberate the virus from the infected cells. Viral replicase genes have also been present in novel coronavirus with a variable number open reading frame (ORF), which codes essential proteins for replicating the virus, the formation of nucleocapsid and spikes, etc..^1^ About 2/3rd of the RNA of SARS-CoV-2 principally placed in the 5′-terminal in the first ORF translates two polyproteins which code for 16 different non-structural proteins (NSP);^24^ on the other hand, remaining ORFs codifies other eight accessory proteins interceding with the nonspecific defense mechanisms.^2,25^ Also, there are some intraviral protein-to-protein communications with these proteins, ultimately leading to such complicated situations.

**Figure 1.**
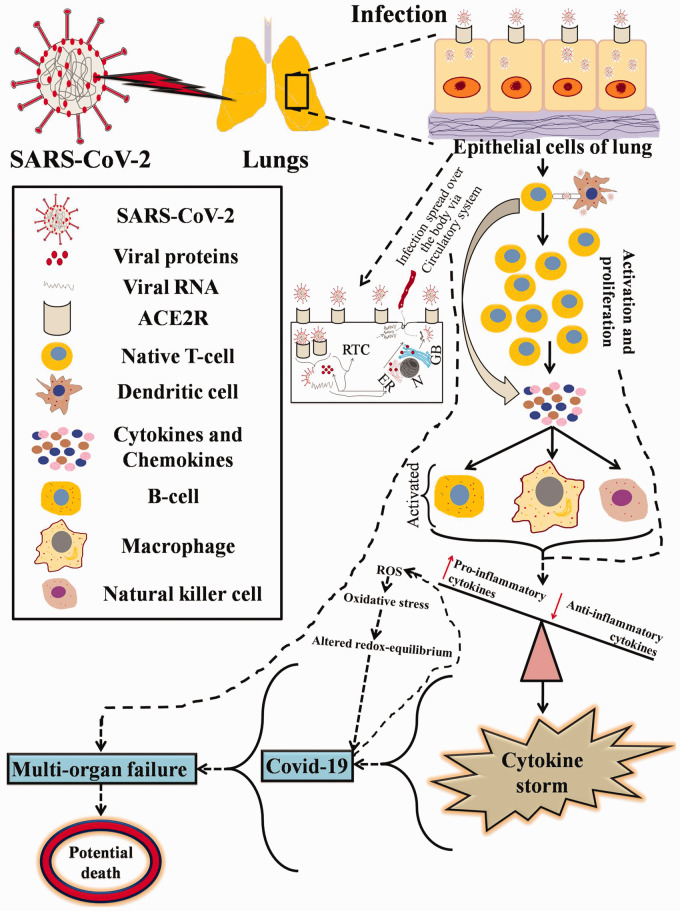
Pathogenicity of SARS-CoV-2-mediated infection (the red upward arrow indicates increase and red downward arrow indicates decrease. (A color version of this figure is available in the online journal.) RTC: replication-transcription complex; ER: endoplasmic reticulum; N: nucleus; GB: Golgi bodies).

The pathogenesis of the SARS-CoV-2 depends upon various factors, including the interaction with the receptor, responsiveness of the immune system, and genetic factors. The pathogenicity of the SARS-CoV-2 has occurred in multiple stages; primarily when the SARS-CoV-2 enters the body via nasal cavity or mouth and ultimately goes to the lungs, the S1 subunit of S protein ties-up with the ACE2R on type II alveolar epithelial cells. It leads to the conformational alteration of the S2 subunit. It stimulates the association between heptad repeat (HR) 1 and HR2 domains to construct 6-HB, which assists the cell membrane and viral membrane in adjacent positions a fusion. This fusion induced damage of the lungs which is the primary infection site by SARS-CoV-2. The lungs’ infection site thereby induced activation of CD4+ and CD8+ T cells; alteration of pro-inflammatory and anti-inflammatory affects lungs and cytokines levels, increased chemokines, lymphocytopenia, increased production of IgM and IgG, cytotoxicity of the lymphocytes, severe organ dysfunction due to abnormal regulation of host response to SARS-CoV-2 infection. On the other hand, lung tissue inflammation, acute respiratory distress syndrome, inflammation-induced lung injury, failure of the respiratory system and other organs, and death. Moreover, the virus SARS-CoV-2 also has harmful impacts on other organs such as the brain to cause cerebral damage and neurologic disturbances, or cardiomyocytes of the heart with escalated angiotensin II. Additionally, it could generate oxygen-free radicals by hypoxia. Hypoxia-reperfusion can also lead to cardiac injury, injury to renal tubular cells of kidney and hepatocytes, or bile duct cells of the liver. All these events can eventually induce hepatocellular anomalies via binding on ACE2R, respectively.^26^ The spike proteins of the SARS-CoV-2 recognize the receptors on the cell surface to penetrate the host cells just like the HIV GP 120 protein does, but the only difference is the specificity of the receptor. In the case of SARS-CoV-2, it is ACE2R to penetrate mucosal epithelial cells and cluster of differentiation four receptors (CD4R) for HIV to penetrate CD4+ T the cells; Figure 1 shows the overall mechanism pathogenicity of SARS-CoV-2 and how it invades into the epithelial cells of the lung.^27^ The mechanism of cellular entry of the SARS-CoV-2 via ACE2R by fusion with the cell membrane and then it ultimately liberated its RNA. Further, in the endoplasmic reticulum or in the cytoplasm, it synthesizes viral proteins, and some of the viral protein (i.e. membrane glycoproteins, spike proteins, envelope proteins, and nucleocapsid protein) and RNA ultimately construct a replication complex to enhance the synthesis of RNA, or it increases the copy numbers of RNA. Next, it is packaged with the Golgi apparatus and then released into the circulatory system. Therefore, it is common speculation that via the circulatory system or blood flow for spreading the virus not only affects lungs but also poses a potential threat to the other important structure of the human body.^27^ Additionally, ACE2R plays a crucial role in the body, and it has a high affinity to Angiotensin II type 1 and 2 receptors with diverse physiological functions. ACE2R is expressed in various organs, tissues in the human body controlling renal, reproductive, and cardiovascular function. Therefore, some reports stated the knockdown of this receptor, application of ACE2R inhibitor, or silencing the angiotensin-converting enzyme 2 can reduce the infection of the SARS-CoV-2. Still, the aftereffect in the body is very unfavorable as it regulates different physiological functions.^28,29^ Thus, in the present review, we propose using the CRISPR/Cas system as an alternative way of an antiviral management strategy against muscular SARS-CoV-2 without or fewer side effects.

## CRISPR/Cas to combat against SARS-CoV-2

The world’s present pandemic situation is because of the SARS-CoV-2; patients with SARS-CoV-2 infection and comorbidities such as obesity, diabetes, non-alcoholic fatty liver disease, cardiovascular disorder, etc. strengthen the mortality rate.^30^ On the other hand, failure of effective treatments, or lack of effective drugs or drugs with lots of adverse effects, and absence of vaccines make it a serious health concern and ultimately leads to a pandemic situation. The CRISPR/Cas system has concentrated on trace and degrades the intracellular viral mRNAs and genome. In contrast, the other common vaccines or drugs depend on humans’ immune systems to trace the viral proteins and some components to reduce the virus’s entrance into the cells.^1^ In this context, the present review suggests an alternative antiviral approach of management to combat this pandemic situation by using the CRISPR/Cas system.

Accumulating evidence suggests that the CRISPR/Cas system has antiviral potential to treat virus-mediated diseases via genomic editing or degrading the DNA or RNA sequences to stop the viral replication and translation. Although, in the present scenario, COVID-19 is not a genetic abnormality; where SARS-CoV-2 is the master player of such infection which belongs to the family of RNA viruses, enters the epithelial cells of the human to infect them, liberates its RNA genome into the cytoplasm, and then synthesizes to increase the copy number of viral RNA to construct a new virus to infect adjacent cells.^31^ However, to combat the novel coronavirus, it is necessary to utilize RNA research, which may explain an important way of developing an antiviral strategy to fight against the virus and terminate the infections. On the CRISPR-based other hand, DNA is the genetic material transcribes into the RNA in the human body, but when the DNA is in the cell’s nucleus, the genetic information is carried by RNA to the cell. Moreover, COVID-19 caused by SARS-CoV-2 is also a sneaky RNA virus that causes the human body’s protein synthesis machinery to fault it for RNA constructed by our DNA. Once the SARS-CoV-2 infects the human body, it continuously liberates RNA; long viral proteins are produced from the human body’s hijacked cells, which are the great source of virus, and it fails to heal our immune system to protect us from SARS-CoV-2 infections. After that, the virus rapidly spreads all over the body via body fluids and infects other humans through the fluidic medium.^32^

In this context, as an alternative antiviral strategy of management to fight against the SARS-CoV-2-mediated infection, the present review proposes the CRISPR/Cas system, the Cpf1 and C2c2/Cas13 for editing of genes in case of RNA virus or SARS-CoV-2.^34^ An earlier study demonstrated that the Cas9 targets endogenous mRNA and thereby controls the expression of target genes.^33^ Therefore, it can be speculated that to manipulate the SARS-CoV-2-like RNA virus or host factor’s mRNA, an RNA-induced RNA targeting system could be an alternative antiviral strategy of management. Moreover, till now, lack of effective antiviral strategy of management to combat against RNA virus such as Dengue Virus (DENV) and Zika Virus (ZIKV), West Nile Virus (WNV), Influenza Virus, Ebola Virus (EBOV), SARS-CoV, MERS-CoV are the hottest topic of current research field.^34^ To survive this, it is important to understand the host's interaction with the virus. CRISPR/Cas system-based work has now gained importance to uncover the underlying mechanism and knockdown the target genes to inhibit viral replication and infection.^34^ RNA targeting the CRISPR system could be used to silence the transcriptional genetic factors. Recently, CRISPR (type VI)/Cas13 system has been suggested as an important tool for gene silencing because there are various important Cas13 target sites that exist in the genome of the single-stranded RNA (ssRNA) virus, and Cas13 is an efficient antiviral against different ssRNA viruses and lastly it acts as programmable scissors to cut the complementary RNA to its crRNA. Moreover, recent reports suggested that Cas13 has significant antiviral activity against some ssRNA viruses; it has also well documented that the antiviral activity of the Cas13 assisted restriction viral expression and readout (CAVER) in which Cas13 identifies and destroys the viral RNA.^36^

Additionally, the RNA-guided as well as targeting manipulation of the gene via the Cas13 enzymes permit the sorting out of the target genes; this unique characteristic feature of Cas13 may be effective therapeutic tools in genetic engineering-based COVID-19 treatment by eliminating the target sequences in the genome; moreover, a recent study suggested that Cas13 fight against the transcriptional as well as splicing-associated RNA binding protein and furthermore they also used the guide RNA as a tool for further analysis.^35,36^

Besides the antiviral application of Cas13 in targeting and knocking down viruses can also be utilized to evaluate as tool for replication, localization, and evolution of virus.^37^ This approach is well supported by earlier studies which reveal that the catalytically dead mutants of Cas13 (Cas13d) can be mainly utilized to investigate the localization of viral RNA, and the RNA-editing ability of Cas13d fusion proteins could be an alternative marker to recognize the viral polymorphisms functionally.^38–40^ Therefore, the CRISPR/Cas13 system could be an antiviral tool for diagnosis and treatment of virus-mediated infections. By these tools, researchers can accurately reach the research’s peak and show the way to a quick cure against the virus.

 Very recently, a group of researchers of Stanford University stated that CRISPR/Cas13 specifically targets and knockdown viral RNA sequences liberated from the SARS-CoV-2 to fix the COVID-19; they suggested that CRISPR-based technology inhibits viral infections.^31^ Prophylactic antiviral CRISPR in human cells (PAC-MAN) can elicit strong viral degradation by neutralizing the SARS-CoV-2 and inhibiting intracellular replication; the enzyme Cas13 is a viral degrading enzyme that degrades nucleic acid sequences in the genome of coronavirus. PAC-MAN has now been suggested to kill the virus-mediated infections via potentially degrading the RNA of the sequence from live influenza A virus and SARS-CoV-2 of humans. PAC-MAN uses CRISPR/Cas13 for the inhibition of the SARS-CoV-2. It can be hypothesized from the earlier report that when SARS-CoV-2 infects the cell of human body, it releases RNA and thereby more copies of viral mRNA and genomes are produced, and the Cas13d tool acts as an inhibitor to attenuate the replication of the virus and function too via immediately cut the viral positive-sense RNA of SARS-CoV-2.^32^ A recent report suggested that the CRISPR/Cas13 system reduced the viral load of epithelial cells of the lungs of humans infected with the H1N1 strain of the flu and SARS-CoV-2. Therefore, we can speculate that it might be feasible from the pharmacological standpoint to develop any such drug which is approved to use in the case of a human trial.^31^ A group of researchers and scientists already found that two highly conserved zones of the genome of SARS-CoV-2 can be suitable to be directed by PAC-MAN as an antiviral strategy to inhibit the replication and spreading of the virus by RNA-dependent RNA polymerase gene (RdRP gene) in the open reading frame (ORF1a/b) region; it regulates the multiplication of coronavirus and nucleocapsid (N) gene (at 3ʹ end of the genome) which are responsible for the viral packaging of capsid protein.^31^ Furthermore, another report stated that a group of researchers designed 10,333 guide RNAs protecting from to target specifically 10 peptide coding regions of the virus RNA genome which caused the COVID-19 including ORF1a/b and the spike gene also.^41^

The CRISPR/Cas13 and PAC-MAN also acts as antiviral weapon for attacking the RNA sequences of SARS-CoV-2 to blunt the pandemic situation caused by a novel coronavirus. However, in the context of human trials of CRISPR-based technology for any viral disease-related treatment, must preserve caution vis-a-vis inflammatory response and other negative clinical consequences. Moreover, we have to mark the hurdles that need to be overcome before the use of the technology in the clinics as an alternative approach to antiviral therapy. It is more promising than the validation of CRISPR-based technology in preclinical organoid models or animal models, and terminal clinical trials would evidently be an immense and laborious task.

## Barriers and restrictions of the use of CRISPR/Cas system and PAC-MAN as antiviral strategy against SARS-CoV-2 infection

**Figure 2.**
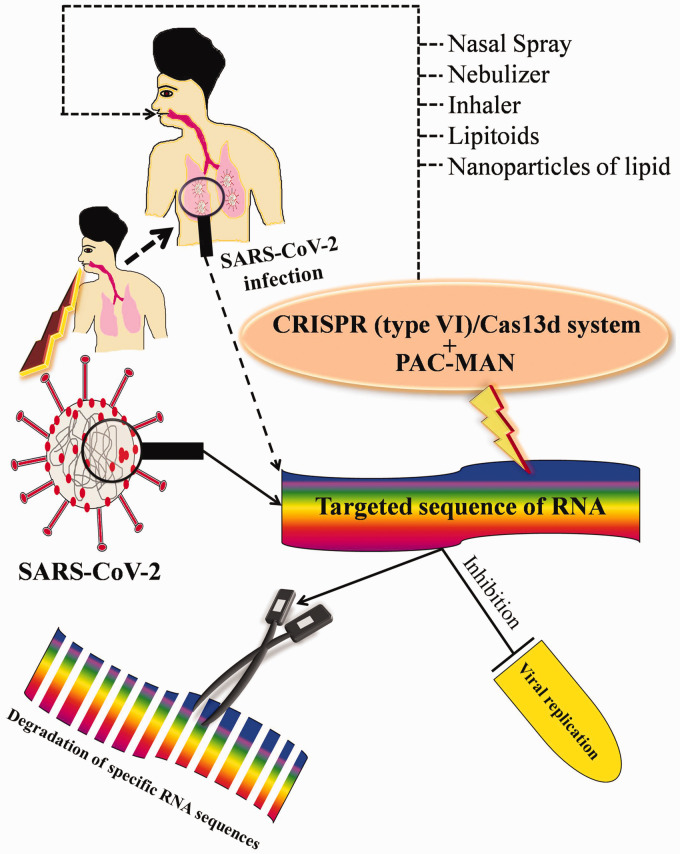
Hypothetical target mechanism of gene manipulation to combat against deadly SARS-CoV-2-mediated infection. The schematic diagram represents the possible mechanism of gene manipulation by CRISPR/Cas13d and PAC-MAN via degradation of specific target sequence of SARS-CoV-2 to inhibit the viral replication to protect the human health. (A color version of this figure is available in the online journal.)

As CRISPR/Cas-based technology is more recent than the other genomic editing tools such as zinc-finger nucleases, transcription activator-like effector nucleases are more specific and highly efficient than others. Although it has some limitations, majorities of the study have been executed in *in vitro* models and lack of *in vivo* data from animal models by using the CRISPR/Cas system. Therefore, it fails to explain its therapeutic potential in humans. Moreover, earlier reports have demonstrated that CRISPR/Cas-based technology incited virus escape mutants and developed a higher rate of virus pathogenicity. Although this issue has been sorted out by the application of multiple gRNAs directed adjacent to various genomic sites, still in some cases, there are some issues.^9,42^ On the contrary, delivery of Cas9:guide RNA (gRNA) is an important therapeutic component to confirm the success and potentiality of consideration of viral vectors.^16,43^ Therefore, to develop a safe and efficient antiviral strategy against any viral disease via CRISPR/Cas system, it is very important for the success of the system *in vivo* models, clinics, targeting of organs, tissues, and cells of humans yet to be a challenge. Furthermore, there are some known provocations that can stimulate the reactivation of the virus and subsequent spreading of the replication of the virus, which causes serious problems like pandemics all over the world. Additionally, the PAC-MAN strategy limitation is the delivery method in the body; it can be delivered through chemical polymers or nanoparticles of lipid.^44–47^ Moreover, liposome-mediated delivery is another option also such as lipitoids, HEDGES platform.^48,49^ On the other hand, it can deliver via ribonucleoprotein complex with Cas13d protein tagged renin-angiotensin with crRNA,^47,50^ engineered amphiphilic peptides.^51^ Additionally, the advancement of gene therapy-based approaches with safe and efficient non-viral delivery systems such as self-assembled peptide-poloxamine nanoparticles could also be an alternative one.^52^ Figure 2 shows a hypothetical target pathway of gene manipulation and an alternative strategy of management to combat deadly SARS-CoV-2 mediated infection to weaken the COVID-19 pandemics. Therefore, it can be assumed that all these antiviral techniques can be given to patients by using a nebulizer or a nasal sprayer. Further studies are required to identify the technology’s barriers or disadvantages before initiating it directly to fight against novel coronavirus. However, it is crucial to recognize and investigate the disadvantageous impacts of the use of single-guide RNAs (sgRNAs) or CRISPR/Cas system or PAC-MAN on the physiology of the host. However, if we can limit the adverse effect on the integrity of the genome and transcriptome profiles, then PAC-MAN and CRISPR/Cas13d system will be an attractive and useful therapeutic strategy against SARS-CoV-2-mediated infection via inhibition of viral replication and function by cleavage of the specific sequences of the target RNA of SARS-CoV-2.

## Conclusions

As a tool of molecular biology and genetic engineering, CRISPR/Cas13d along with PAC-MAN acts as an antiviral tool with high flexibility and specificity for the target disease to inhibit RNA virus-mediated infection. Further studies are required to evaluate the success rate and safety of this antiviral tool-based technology to fix the SARS-CoV-2-mediated infection in animals before its potential therapeutic use to SARS-CoV-2-infected patients. If it proves to be effective, it could be used as a weapon to prevent SARS-CoV-2-induced infections worldwide as a pattern of the advancement of medical science via knockdown or degradation of specific RNA sequence in SARS-CoV-2 and associated comorbidities.
